# Microwave-Assisted Synthesis of some Novel Azoles and Azolopyrimidines as Antimicrobial Agents

**DOI:** 10.3390/molecules22030346

**Published:** 2017-02-23

**Authors:** Sobhi M. Gomha, Thoraya A. Farghaly, Yahia Nasser Mabkhot, Mohie E. M. Zayed, Amany M. G. Mohamed

**Affiliations:** 1 Department of Chemistry, Faculty of Science, Cairo University, Giza 12613, Egypt; s.m.gomha@gmail.com (S.M.G.); amaaniq21@yahoo.com (A.M.G.M.); 2 Department of Chemistry, Faculty of Applied Science, Umm Al-Qura University, Makkah-Al-mukkarramah 21514, Saudi Arabia; 3 Department of Chemistry, College of Science, King Saud University, P.O. Box 2455, Riyadh-11451, Saudi Arabia; 4 Chemistry Department, Faculty of Science, King Abdulaziz University, Jeddah B.O. 208203, Saudi Arabia, mohiem@yahoo.com

**Keywords:** thiophenes, pyrazoles, thiazoles, antimicrobial activity, microwave irradiation

## Abstract

In this study, new derivatives of pyrazole, isoxazole, pyrazolylthiazole, and azolopyrimidine having a thiophene ring were synthesized under microwave irradiation. Their pharmacological activity toward bacteria and fungi inhibition was screened and compared to the references *Chloramphenicol* and *Trimethoprim*/*sulphamethoxazole*. The antimicrobial results of the investigated compounds revealed promising results and some derivatives have activities similar to the references used.

## 1. Introduction

Five-membered heterocyclic ring systems are very significant class of compounds, not only due to their abundance in nature, but also for their chemical and biological value. Thiophene derivatives have been fully-known for their therapeutic applications. They possess antihypertensive [1], antimicrobial [2], diabetes mellitus [3], antiviral [4], analgesic and anti-inflammatory [5], and antitumor activities [6,7]. Pyrazoles and thiazoles exist in many naturally occurring substances and representing an interesting array of azole compounds. They have a wide range of biological activities as for example, anti-inflammatory [8,9], antimicrobial [10,11,12,13], Akt kinase inhibitive [14], anticonvulsant [15], and antitumor activities [16]. On the other hand, microwave-assisted organic synthesis is a tool by which we can achieve goals in a few minutes with high yield as compared to conventional heating [17,18,19,20,21]. Motivated by these findings, and in continuation of our ongoing research program dealing with the synthesis of bioactive heterocyclic ring systems [22,23,24,25,26], we were encouraged to synthesize heterocyclic having thiophene incorporated pyrazole, thiazole, and/or pyrimidine derivatives under microwave irradiation to investigate their antimicrobial activity.

## 2. Results and Discussion

### 2.1. Synthesis

**Scheme 1:**
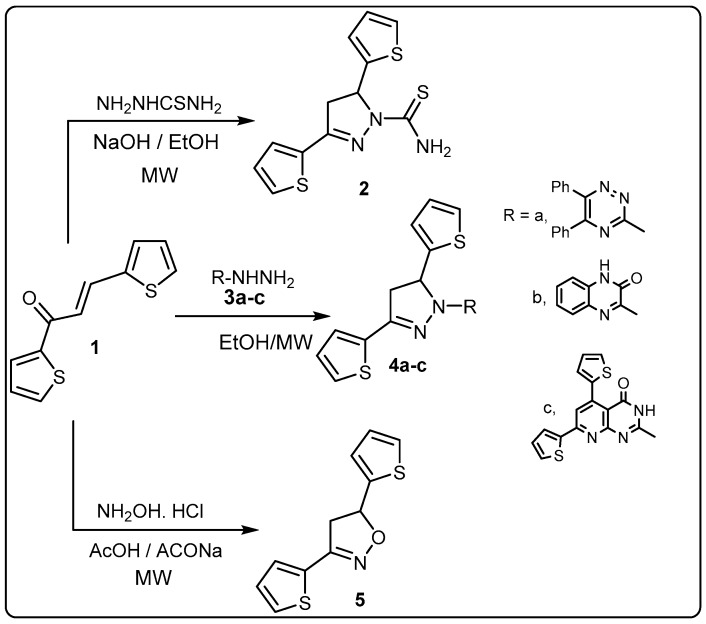
Synthesis of pyrazoline derivatives **2**, **4a**–**c**, and **5**.

1,3-Di(thiophen-2-yl)prop-2-en-1-one **1** was cyclized with different types of nitrogen nucleophiles, namely, thiosemicarbazide, hydrazine derivatives **3a**–**c**, and hydroxylamine hydrochloride which afforded pyrazole derivatives **2**, **4a**–**c** and isoxazole derivative **5**, respectively (Scheme 1). The previous reactions were carried out under conventional heating and under microwave irradiation as shown in Table 1. The heating under microwave was more efficient than thermal heating as it reduced the reaction time and increased the product yields in all cases.

**Scheme 2:**
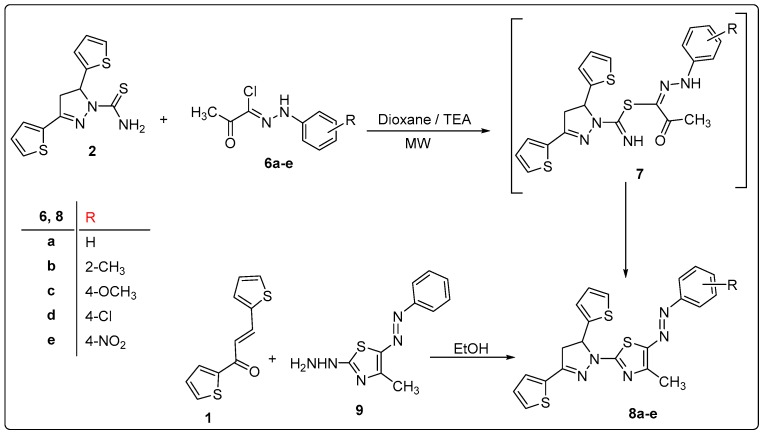
Synthesis of arylazothiazole derivatives **8a**–**e**.

It was reported that pyrazolylthiazole derivatives have a wide range of biological activities such as antimicrobial [27], anti-inflammatory [27], hypotensive [28], and antitumor activities [29]. So we became interested in synthesizing the pyrazolylthiazole derivatives from the reaction of 1-thiocarbamoyl-3,5-di-(2-thienyl)-2-pyrazoline **2** with hydrazonoyl chlorides. Thus, conventional heating or microwave irradiation of mixture of carbothioic acid amide derivative **2** and 2-oxo-*N*-arylpropanehydrazonoyl chloride **6a**–**e** in dioxane in the existence of a base catalyst yielded in each case only one isolated product (Scheme 2). The spectroscopic information confirmed the reaction products **8a**–**e**. For example, the mass spectra of the isolated products **8a**–**e** displayed the expected molecular ion. Also, all derivatives **8a**–**e** showed in their ^1^H-NMR spectra the characteristic signals for CH_3_, H-5, and CH_2_ (see experimental part). The structure of products **8** was further supported by an alternative synthesis. Thus, reaction of compound **1** with 2-hydrazinyl-4-methyl-5-(phenyldiazenyl)thiazole **9** under reflux in ethanol led to the formation of product **8a** (Scheme 2).

**Scheme 3:**
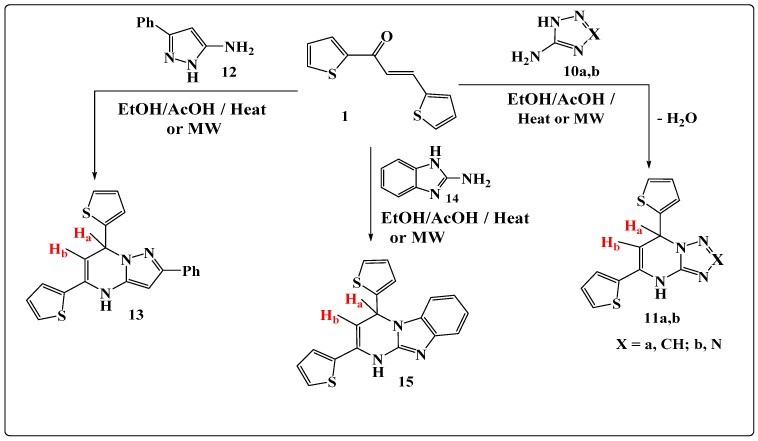
Synthesis of azolopyrimidine derivatives **11a**,**b**, **13** and **15**.

Azolopyrimidines **11a**,**b**, **13**, and **15** were prepared via the reaction of chalcone **1** with heterocyclic amines **10a**,**b**, **12**, and **14** in ethanol in the presence of catalytic amount of AcOH using both thermal heating and microwave irradiation for comparison (Scheme 3). Similar to the preparation of compounds **2**, **4**, **5**, and **8a**–**e**, the use of microwave irradiation was more effective in the synthesis of azolopyrimidines as illustrated in Table 1. The structure of compounds **11a**,**b**, **13**, and **15** was confirmed by different spectroscopic techniques like IR, ^1^H-NMR, mass, and elemental analysis. The IR spectra of **11a**,**b**, **13**, and **15** revealed the absence of any absorption bands for carbonyl group in addition to the presence of absorption band for NH group at 3402–3429 cm^−1^. The ^1^H-NMR spectra of **11a**, as example, showed three characteristic signals for the two CH-pyrimidine, triazole-H, and NH at δ 5.14 (d, *J* = 4 Hz, 1Ha, CH-pyrimidine), 6.20 (d, *J* = 4 Hz, 1Hb, CH-pyrimidine), 8.45 (1H, s, triazole-H), 8.73 (s, br, 1H, NH).

### 2.2. Antimicrobial Activity

In vitro antimicrobial screening of compounds **2**, **4a**–**c**, **5**, **8a**–**e**, **11a,b**, **13**,and **15** prepared in the study was carried out using cultures of two fungal strains *Aspergillus niger* (ATCC) (ASP) and *Candida albicans* (ATCC10231) (CA), as well as three bacteria species, namely, Gram positive bacteria, *Staphylococcus aureus* (ATCC 29213) (SA), and *Bacillus subtilus* (ATCC 6051) (BS) and the Gram negative bacteria is *Escherichia coli* (ATCC 25922) (EC). *Chloramphenicol* and *Trimethoprim*/*sulphamethoxazole* antibacterial agents were used as references to evaluate the potency of the examined compounds under the same conditions. The activity was investigated by measuring the diameter of inhibition zone (IZD) in mm ± standard deviation beyond well diameter (6 mm) generated on a range of environmental and clinically pathogenic microorganisms (gram-positive and gram-negative bacteria and fungi) utilizing (0.1 g/mL) concentration of tested samples and the outcomes are portrayed in Table 2. For the antifungal activity: All tested compounds were inactive against *Aspergillus niger* (ATCC) (ASP) while, compounds **4c**, **8c**, and **11b** have excellent activity against *Candida albicans* (ATCC 10231) (CA) with inhibition zones 23, 24, and 25 respectively. For the antibacterial activity: it was found that Gram positive bacteria are more sensitive to the tested compounds especially SA rather than *BS* as five compounds **2**, **4c**, **8b**, **8d**, and **15** have potent activity against *SA* while for *BS* only compounds **4a** and **4c** showed good activity. In the case of Gram negative activity with EC, two derivatives **2** and **8c** revealed higher activity. The used solvent DMSO concentration did not exhibit any influence on bacteria or fungi.

## 3. Materials and Methods

### 3.1. General Experimental Procedures

Melting points were measured with an IA 9000-series digital melting-point apparatus (Bibby Sci. Lim. Stone, Staffordshire, UK). Solvents were generally distilled and dried by standard literature procedures prior to use. IR spectra were recorded in potassium bromide discs on FTIR 8101 PC infrared spectrophotometers (Shimadzu, Tokyo, Japan). NMR spectra were recorded on a Mercury VX-300 NMR spectrometer (Varian, Inc., Karlsruhe, Germany) operating at 300 MHz (^1^H-NMR) and run in deuterated dimethylsulfoxide (DMSO-*d*_6_). Chemical shifts were related to that of the solvent. Mass spectra were recorded on a Shimadzu GCeMS-QP1000 EX mass spectrometer (Tokyo, Japan) at 70 eV. Microwave reactions were performed with a Millstone Organic Synthesis Unit with a touch control terminal (MicroSYNTH, Giza, Egypt) and a continuous focused microwave power delivery system in a pressure glass vessel (10 mL) sealed with a septum under magnetic stirring. The temperature of the reaction mixture was monitored using a calibrated infrared temperature control under the reaction vessel, and control of the pressure was performed with a pressure sensor connected to the septum of the vessel. Elemental analyses were carried out at the Microanalytical Centre of Cairo University, Giza, Egypt. Compounds **10a**,**b**, **12**, and **14** were purchased from Sigma-Aldrich and utilized as it is without previous treatments. Compounds **1**, **2**, **6a**–**e**, and **9** were prepared as previously reported in the respective literature [30,31,32].

### 3.2. Synthesis of Pyrazoline Derivatives ***4a***–***c***

Method A: A mixture of chalcone **1** (0.220 g, 1 mmol) and hydrazine derivative (1 mmol) in ethanol (20 mL) in the presence of catalytic drops of acetic acid was refluxed for 3–5 h (monitored by TLC). The reaction mixture was poured into water and the solid product was collected by filtration followed by washing with ethanol. The crude products were then recrystallized from ethanol to give pure pyrazolines **4a**–**c**, respectively.

Method B: Repetition of the same reactions of method A with heating in a microwave oven at 500 W and 120 °C for a period of time. The reaction mixture was treated similar to method A to obtain compounds **4a**–**c**. Compounds **4a**–**c** with their physical constants and spectral data are depicted as shown below:

*3-(3,5-Di(thiophen-2-yl)-4,5-dihydro-1H-pyrazol-1-yl)-5,6-diphenyl-1,2,4-triazine* (**4a**). Brown solid, m.p. 187–189 °C; IR: 3083, 2926 (C-H), 1593 (C=N) cm^−1^; ^1^H-NMR (300 MHz, DMSO-*d*_6_): δ 3.05 (dd, 1H, H_A_, *J* = 17.2, 6.1 Hz), 4.13 (dd, 1H, H_B_, *J* = 17.2, 12.0 Hz), 6.20 (dd, 1H, H_X_, *J* = 12.0, 6.1 Hz), 7.18–8.24 (m, 16H, Ar-H); MS, *m*/*z* (%) 465 (M^+^, 8), 316 (34), 222 (38), 105 (100), 77 (72), 64 (80). Anal. Calcd. For C_26_H_19_N_5_S_2_ (465.11): C, 67.07; H, 4.11; N, 15.04; found: C, 66.87; H, 4.24; N, 14.90.

*3-(3,5-DI(thiophen-2-yl)-4,5-dihydro-1H-pyrazol-1-yl)-3,4-dihydroquinoxalin-2(1H)-one* (**4b**). Brown solid, m.p. 170–172 °C; IR: 3435, 3158 (2NH), 3048, 2966 (C-H), 1596 (C=N) cm^−1^; ^1^H-NMR (300 MHz, DMSO-*d*_6_): δ 3.05 (dd, 1H, H_A_, *J* = 17.2, 6.1 Hz), 4.10 (dd, 1H, H_B_, *J* = 17.2, 12.0 Hz), 6.18 (dd, 1H, H_X_, *J* = 12.0, 6.1 Hz), 7.04–7.99 (m, 10H, Ar-H), 11.88 (s, br, 1H, NH); MS, *m*/*z* (%) 378 (M^+^, 5), 274 (28), 153 (70), 77 (65), 43 (100). Anal. Calcd. For C_19_H_14_N_4_OS_2_ (378.47): C, 60.30; H, 3.73; N, 14.80; found: C, 60.03; H, 3.92; N, 14.52.

*2-(3,5-Di(thiophen-2-yl)-4,5-dihydro-1H-pyrazol-1-yl)-5,7-di(thiophen-2-yl)pyrido[2,3-d]pyrimidin-4(3H)-one* (**4c**). Brown solid, m.p. 188–190 °C; IR: 3435, 3158 (2NH), 3048, 2966 (C-H), 1596 (C=N) cm^−1^; ^1^H-NMR (300 MHz, DMSO-*d*_6_): δ 3.07 (dd, 1H, H_A_, *J* = 17.2, 6.1 Hz), 4.15 (dd, 1H, H_B_, *J* = 17.2, 12.0 Hz), 6.16 (dd, 1H, H_X_, *J* = 12.0, 6.1 Hz), 6.92–7.75 (m, 12H, Ar-H), 7.98 (s, 1H, pyridine-H_4_), 8.63 (s, br, 1H, NH); MS, *m*/*z* (%) 543 (M^+^, 14), 426 (50), 330 (49), 153 (83), 64 (100), 43 (68). Anal. Calcd. For C_26_H_17_N_5_OS_4_ (543.03): C, 57.44; H, 3.15; N, 12.88; found: C, 57.58; H, 3.10; N, 12.63.

### 3.3. 3,5-Di(thiophen-2-yl)-4,5-dihydroisoxazole *(**5**)*

Method A: A mixture of chalcone **1** (0.220 g, 1 mmol), hydroxylamine. HCl (0.069 g, 1 mmol), and anhydrous sodium acetate (0.3 g) in acetic acid (20 mL) was stirred at room temperature for 6 h. The formed solid was filtered, washed with water, and crystallized from dioxane to give isoxazoline derivative **5**.

Method B: The above reaction of chalcone **1** and hydroxylamine with the same quantity in method A were heated under microwave irradiation at 500 W and 150 °C for 10 min. The reaction mixture was treated similarly to method A to obtain compounds **5** as yellow solid; m.p. 212–214°C; IR: 3091, 2922 (C-H), 1593 (C=N) cm^−1^; ^1^H-NMR (300 MHz, DMSO-*d*_6_): δ3.09 (dd, 1H, H_A_, *J* = 17.2, 6.1 Hz), 4.13 (dd, 1H, H_B_, *J* = 17.2, 12.0 Hz), 6.08 (dd, 1H, H_X_, *J* = 12.0, 6.1 Hz), 7.00–8.23 (m, 6H, Ar-H); MS, *m*/*z* (%) 335 (M^+^, 24), 152 (65), 83 (100), 70 (21). Anal. Calcd. for C_11_H_9_NOS_2_ (235.01): C, 56.14; H, 3.85; N, 5.95; found: C, 56.03; H, 3.72; N, 5.74.

### 3.4. Synthesis of 2-(3,5-Di(thiophen-2-yl)-4,5-dihydro-1H-pyrazol-1-yl)-4-methyl-5-(aryldiazenyl)thiazoles ***8a***–***e***

Method A: A mixture of 3,5-di(thiophen-2-yl)-4,5-dihydro-1*H*-pyrazole-1-carbothioamide **2** (0.293 g, 1 mmol) and the appropriate hydrazonoyl halides **6a**–**e** (1 mmol) in dioxane (20 mL) containing TEA (0.5 mL) was refluxed for 6–10 h (monitored by TLC), allowed to cool and the solid formed was filtered off, washed with ethanol, dried, and recrystallized from dimethylformamide to give **8a**–**e**.

Method B: Repetition of the same reactions of method A with heating in microwave oven at 500 W and 150 °C for a period of time gave products identical in all respects with those separated from method A.

*2-(3,5-Di(thiophen-2-yl)-4,5-dihydro-1H-pyrazol-1-yl)-4-methyl-5-(phenyldiazenyl)-thiazole* (**8a**). Red solid, m.p. 164–166 °C; IR:2919 (C-H), 1603 (C=N) cm^−1^; ^1^H-NMR (300 MHz, DMSO-*d*_6_): δ 2.58 (s, 3H, CH_3_), 3.07 (dd, 1H, H_A_, *J* = 17.2, 6.1 Hz), 4.17 (dd, 1H, H_B_, *J* = 17.2, 12.0 Hz), 6.21 (dd, 1H, H_X_, *J* = 12.0, 6.1 Hz), 7.00–7.84 (m, 11H, Ar-H); MS, *m*/*z* (%) 435 (M^+^, 5), 339 (14), 205 (50), 75 (42), 50 (100). Anal. Calcd. for C_21_H_17_N_5_S_3_ (435.06): C, 57.90; H, 3.93; N, 16.08; found: C, 57.74; H, 3.77; N, 15.82.

*2-(3,5-Di(thiophen-2-yl)-4,5-dihydro-1H-pyrazol-1-yl)-4-methyl-5-(o-tolyldiazenyl)-thiazole* (**8b**). Red solid, m.p. 122–124 °C; IR: 2921 (C-H), 1600 (C=N) cm^−1^; ^1^H-NMR (300 MHz, DMSO-*d*_6_): δ 2.04 (s, 3H, CH_3_), 2.60 (s, 3H, CH_3_), 3.09 (dd, 1H, H_A_, *J* = 17.2, 6.1 Hz), 4.19 (dd, 1H, H_B_, *J* = 17.2, 12.0 Hz), 6.22 (dd, 1H, H_X_, *J* = 12.0, 6.1 Hz), 6.93–7.79 (m, 10H, Ar-H); MS, *m*/*z* (%) 449 (M^+^, 18), 218 (12), 110 (48), 91 (100), 65 (52). Anal. Calcd. for C_22_H_19_N_5_S_3_ (449.08): C, 58.77; H, 4.26; N, 15.58; found: C, 58.52; H, 4.08; N, 15.46.

*2-(3,5-Di(thiophen-2-yl)-4,5-dihydro-1H-pyrazol-1-yl)-5-((4-methoxyphenyl)diazenyl)-4-methylthiazole* (**8c**). Red solid, m.p. 143–145 °C; IR: 2923 (C-H), 1602 (C=N) cm^−1^; ^1^H-NMR (300 MHz, DMSO-*d*_6_): δ 2.56 (s, 3H, CH_3_), 3.04 (dd, 1H, H_A_, *J* = 17.2, 6.1 Hz), 3.83 (s, 3H, OCH_3_), 4.12 (dd, 1H, H_B_, *J* = 17.2, 12.0 Hz), 6.13 (dd, 1H, H_X_, *J* = 12.0, 6.1 Hz), 6.92–7.82 (m, 10H, Ar-H); MS, *m*/*z* (%) 465 (M^+^, 3), 368 (9), 218 (13), 111 (100), 43 (72). Anal. Calcd. for C_22_H_19_N_5_OS_3_ (465.08): C, 56.75; H, 4.11; N, 15.04; found: C, 56.53; H, 4.04; N, 14.86.

*5-((4-Chlorophenyl)diazenyl)-2-(3,5-di(thiophen-2-yl)-4,5-dihydro-1H-pyrazol-1-yl)-4-methylthiazole*(**8d**). Orange solid, m.p. 153–155 °C; IR: 3063, 2922 (C-H), 1605 (C=N) cm^−1^; ^1^H-NMR (300 MHz, DMSO-*d*_6_): δ 2.56 (s, 3H, CH_3_), 3.08 (dd, 1H, H_A_, *J* = 17.2, 6.1 Hz), 4.08 (dd, 1H, H_B_, *J* = 17.2, 12.0 Hz), 6.16 (dd, 1H, H_X_, *J* = 12.0, 6.1 Hz), 6.98–7.85 (m, 10H, Ar-H); MS, *m*/*z* (%) 471 (M^+^+2, 1), 469 (M^+^, 4), 368 (6), 264 (15), 111 (59), 77 (57), 43 (100). Anal. Calcd. for C_21_H_16_ClN_5_S_3_ (469.03): C, 53.66; H, 3.43; N, 14.90; found: C, 53.49; H, 3.40; N, 14.73.

*2-(3,5-Di(thiophen-2-yl)-4,5-dihydro-1H-pyrazol-1-yl)-4-methyl-5-((4-nitrophenyl)-diazenyl)thiazole* (**8e**). Brown solid, m.p. 162–164 °C; IR: 3096, 2920 (C-H), 1590 (C=N) cm^−1^; ^1^H-NMR (300 MHz, DMSO-*d*_6_): δ 2.61 (s, 3H, CH_3_), 3.09 (dd, 1H, H_A_, *J* = 17.2, 6.1 Hz), 4.11 (dd, 1H, H_B_, *J* = 17.2, 12.0 Hz), 6.21 (dd, 1H, H_X_, *J* = 12.0, 6.1 Hz), 6.58–7.95 (m, 10H, Ar-H); MS, *m*/*z* (%) 480 (M^+^, 7), 427 (16), 232 (31), 111 (100), 77 (59), 43 (86). Anal. Calcd. for C_21_H_16_N_6_O_2_S_3_ (480.58): C, 52.48; H, 3.36; N, 17.49; found: C, 52.28; H, 3.19; N, 17.33.

### 3.5. Alternate Synthesis of ***8a***

Equimolar amounts of chalcone **1** (0.220 g, l mmol) and 2-hydrazinyl-4-methyl-5-(phenyldiazenyl)thiazole (**9**) (0.233 g, 1 mmol) in 2-propanol (10 mL), was refluxed for 2 h then cooled to room temperature. The solid precipitated was filtered off, washed with water, dried, and recrystallized from dimethylformamide to give the corresponding product, **8a** which were identical in all aspects (m.p., mixed m.p. and IR spectra) with those obtained from reaction of **2** with **6a** but in 70% yield.

### 3.6. General Method for Synthesis of Compounds ***11a**,**b***, ***13***, and ***15***

Method A: A mixture of chalcone **1** (0.220 g, 1 mmol) and the appropriate heterocyclic amine (**10a**,**b**, **12** or **14**) (1 mmol) in ethanol (20 mL) in the presence of catalytic drops of acetic acid was refluxed for 10–15 h (monitored through TLC). The reaction mixture was poured into water and the solid product was collected by filtration followed by washing with ethanol. The crude product was then recrystallized from EtOH or DMF to give pure products **11a**,**b**, **13**, and **15**, respectively.

Method B: Repetition of the same reactions of method A with heating in microwave oven at 500 W and 150 °C for a period of time gave products identical in all respects with those separated from method A. Compounds **11a**,**b**, **13**, and **15** with their physical constants and spectral data are depicted as shown below:

*5,7-Di(thiophen-2-yl)-4,7-dihydro-[1,2,4]triazolo[1,5-a]pyrimidine* (**11a**). Yellow solid m.p. 241–243 °C (DMF); IR: 3425 (NH), 3091, 2920 (C-H), 1599 (C=N), cm^−1^; ^1^H-NMR: δ 5.14 (d, *J* = 4 Hz, 1Ha, CH-pyrimidine), 6.20 (d, *J* = 4 Hz, 1Hb, CH-pyrimidine), 6.85–8.04 (m, 6H, Ar-H), 8.45 (1H, s, triazole-H), 8.73 (s, br, 1H, NH); MS *m/z* (%): 286 (M^+^, 31), 284 (100), 111 (52), 69 (44). Anal. Calcd. for C_13_H_10_N_4_S_2_ (286.03): C, 54.52; H, 3.52; N, 19.56; found: C, 54.40; H, 3.64; N, 19.51.

*5,7-Di(thiophen-2-yl)-4,7-dihydrotetrazolo[1,5-a]pyrimidine* (**11b**). Yellow solid, m.p. 266–268 °C (DMF); IR: 3402 (NH), 3087, 2924 (C-H), 1636 (C=N), cm^−1^; ^1^H-NMR: δ 5.41 (d, *J*= 4 Hz, 1Ha, CH-pyrimidine), 6.08 (d, *J* = 4 Hz, 1Hb, CH-pyrimidine), 7.16–8.24 (m, 6H, Ar-H), 8.29 (s,br, 1H, NH); MS *m/z* (%): 287 (M^+^, 20), 259 (73), 220 (99), 111 (100), 65 (48). Anal. Calcd. for C_12_H_9_N_5_S_2_ (287.03): C, 50.16; H, 3.16; N, 24.37; found: C, 50.29; H, 3.07; N, 24.39.

*2-Phenyl-5,7-di(thiophen-2-yl)-4,7-dihydropyrazolo[1,5-a]pyrimidine* (**13**)*.* Yellow solid m.p. 218–220 °C (DMF); IR: 3429 (NH), 3095, 3071, 2923 (C-H), 1596 (C=N) cm^−1^; ^1^H-NMR: δ 4.86 (d, *J*= 4 Hz, 1Ha, CH-pyrimidine), 6.14 (d, *J* = 4 Hz, 1Hb, CH-pyrimidine), 6.59 (s, 1H, pyrazole-H), 6.80–8.25 (m, 11H, Ar-H), 8.72 (s,br, 1H, NH); MS *m/z* (%): 361 (M^+^, 27), 359 (100), 228 (16), 111 (49), 77 (63). Anal. Calcd. for C_20_H_15_N_3_S_2_ (361.07): C, 66.45; H, 4.18; N, 11.62; found: C, 66.61; H, 4.09; N, 11.60.

*2,4-Di(thiophen-2-yl)-1,4-dihydrobenzo[4,5]imidazo[1,2-a]pyrimidine* (**15**). Yellow solid, m.p. 230–232 °C (EtOH); IR: 3412 (NH), 3077, 2920 (C-H), 1599 (C=N) cm^−1^; ^1^H-NMR: δ 4.79 (d, *J* = 4 Hz, 1Ha, CH-pyrimidine), 6.12 (d, *J* = 4 Hz, 1Hb, CH-pyrimidine), 6.69–8.27 (m, 10H, Ar-H), 8.49 (s, br, 1H, NH); MS *m/z* (%): 335 (M^+^, 18), 333 (100), 224 (23), 111 (50), 64 (53). Anal. Calcd. for C_18_H_13_N_3_S_2_ (335.06): C, 64.45; H, 3.91; N, 12.53; found: C, 64.68; H, 3.87; N, 12.49.

### 3.7. Biological Activity

#### 3.7.1. Antimicrobial Activity

Antimicrobial activity was determined using the agar disc diffusion assay method as described previously by Hossain et al. [33]. The tested organisms were sub-cultured on Trypticase soya agar medium (Oxoid Laboratories, Corporate, UK) for bacteria and Sabouraud dextrose agar (Oxoid Laboratories, Corporate, UK) for fungi. Chloramphenicol and Trimethoprim/sulphamethoxazole were used as a positive control and DMSO solvent as a negative control. The plates were done in duplicate and average zone of inhibition was calculated. Bacterial cultures were incubated at 37 °C for 24 h while the other fungal cultures were incubated at (25–30 °C) for 3–5 days. Antimicrobial activity was determined by measurement zone of inhibition.

#### 3.7.2. Media Used

Sabouraud dextrose agar: The medium used for isolation of pathogenic yeasts has the following composition (g/L): glucose, 20; peptone, 10; agar, 25 and distilled water, 1 L, pH was adjusted at 5.4. The medium was autoclaved at 121 °C for 15 min.

Trypticase soya agar (TSA): The medium was used to cultivate tested bacteria. It contains (g/L) Tryptone (Pancreatic Digest of Casein) 15.0 g, Soytone (Papaic Digest of Soybean Meal) 5.0 g, Sodium Chloride 5.0 g, Agar 15.0 g, and distilled water 1 L. The medium was autoclaved at 121 °C for 15 min.

## 4. Conclusions

At the end, we have succeeded in the synthesis of new derivatives of pyrazole, isoxazole, pyrazolylthiazole, and azolopyrimidine incorporated with a thiophene ring under microwave irradiation. Different spectroscopic methods and elemental analyses were used to confirm the structures of the newly synthesized compounds. The antimicrobial results of the examined compounds revealed promising results and some derivatives have activities similar to the references used.

## Figures and Tables

**Table 1:** Comparison between conventional heating and microwave irradiation for synthesis of compounds **4a**–**c**, **8a**–**e**, **11a**,**b**, **13**, and **15**.

Compound No.	Reaction Times		Reaction Yields (%)	
	Conventional Methods	Microwave	Conventional Methods	Microwave
**2**	2 h [30]	3 min	66 [30]	84
**4a**	4 h	5 min	70	85
**4b**	5 h	8 min	73	90
**4c**	5h	10 min	69	88
**5**	6 h	10 min	67	82
**8a**	6 h	8 min	74	95
**8b**	8 h	10 min	76	90
**8c**	10 h	12 min	68	92
**8d**	8 h	9 min	72	93
**8e**	10 h	13 min	75	90
**11a**	10 h	12 min	70	89
**11b**	15 h	15 min	60	81
**13**	10 h	15 min	67	88
**15**	13 h	20 min	60	85

**Table 2:** Antimicrobial activity of compounds **2**, **4a**–**c**, **5**, **8a**–**e**, **11a,b**, **13**, and **15** compared to reference drug.

Compound Number	Fungi		Gram Positive Bacteria		Gram Negative Bacteria
	ASP	CA	SA	BS	EC
**2**	NA.	NA.	21	19	23
**4a**	N.A.	N.A.	18	20	15
**4b**	N.A.	20	17	18	18
**4c**	N.A.	23	22	20	17
**5**	N.A.	9	19	12	17
**8a**	N.A	10	12	18	11
**8b**	N.A.	8	21	18	12
**8c**	N.A.	24	18	15	23
**8d**	N.A.	N.A.	22	12	17
**8e**	N.A.	8	18	14	13
**11a**	N.A.	9	N.A.	N.A.	10
**11b**	N.A.	25	19	17	11
**13**	N.A.	12	14	11	8
**15**	N.A.	11	21	19	15
Chloramphenicol	29	25	30	24	29
Trimethoprim/sulphamethoxazole	2.4	13	20	23	24
DMSO	N.A.	N.A.	N.A.	N.A.	N.A.
**High activity** 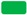 **Moderate activity** 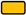 **Low activity** 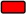 **N.A. (No activity)** 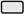					
